# Mental health-related visits in a pediatric emergency department during the COVID-19 pandemic

**DOI:** 10.1186/s12245-021-00387-0

**Published:** 2021-11-04

**Authors:** Arnaud Fernandez, Morgane Gindt, Phillipe Babe, Florence Askenazy

**Affiliations:** 1 Child and Adolescent Psychiatry Department, Children’s Hospitals of Nice CHU-Lenval, 57, Avenue de la Californie, 06200 Nice, France; 2 Université Côte d’Azur, CoBTek, Nice, France; 3 Pediatric Emergency Department, Children’s Hospitals of Nice CHU-Lenval, 57, Avenue de la Californie, 06200 Nice, France

**Keywords:** COVID-19 pandemic, Child and adolescent psychiatry, Mental health, Pediatric emergency department visits

## Abstract

We aimed to describe the epidemiology of all pediatric emergency department visits (focusing on mental health-related visits versus total visits) at the University Children’s Hospital of Nice (France) from 1 January to 31 December 2020 (year of the COVID-19 pandemic) and to compare it with the earlier 3-year period. The increase in mental health-related visits (44.2%) that we observed, while total visits decreased (30.0%), suggests an impact of the pandemic on children’s and adolescents’ mental health.

## Introduction

The year 2020 was marked by the worldwide spread of an emerging virus: severe acute respiratory syndrome coronavirus 2 (SARS-CoV-2).

Comparatively to older adults, children and adolescents are currently less affected by severe and fatal forms of the coronavirus disease 2019 (COVID-19) caused by SARS-CoV-2 infection [1], but are confronted with surrounding anxiety and the worry of being infected [2].

At the University Pediatric Hospital of Nice (UPHN), total visits (including mental health-related visits) to the Pediatric Emergency Department (PED) have been increasing for almost 10 years [3] with relative stability over the last 3 years. Nevertheless, according to literature data, we could hypothesize that we will observe a decrease of all PED visits during the pandemic [4].

The aim of this study was to describe the epidemiology of all PED visits (focusing on mental health-related visits versus total visits) at the UPHN from 1 January to 31 December 2020 and to compare it with the earlier 3-year period.

## Methods

We collected PED visit rates and characteristics at the UPHN. These data were collected over a period ranging from 3 years before the pandemic to the current year of the pandemic (1 January 2017–31 December 2020). We carried out an analysis of the ICM-10 diagnoses and extracted all mental health-related visits (e.g., suicide attempts, suicidal ideation or anxiety). All visits of patients aged 0 to 18 years were included. Then we compared the number of visits to PED in the pandemic period (ranging from 1 January to 31 December 2020) to the number of visits to PED in the 3-year period (mean value) before the pandemic. We analyzed both total and mental health-related visits.

## Results

**Fig. 1:**
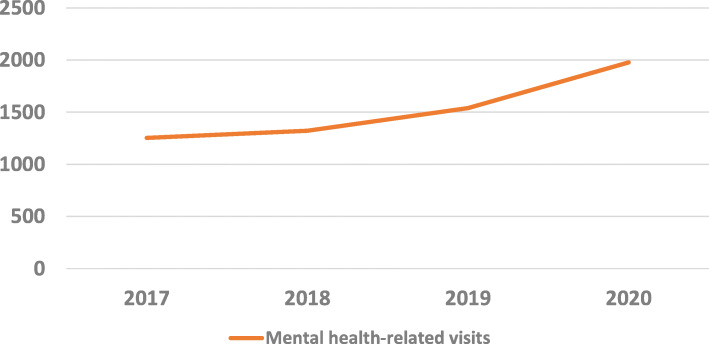
Epidemiology of pediatric emergency department mental health-related visits

The rate of total PED visits in 2020 decreased by 30.0% (42 985 visits) in comparison to the average rate from 2017 to 2019 (61,434 visits in average and 62,429, 60,429 and 61,445 respectively from 2017 to 2019). The rate of mental health-related visits increased by 44.2% (1977 visits) in comparison to the average rate from 2017 to 2019 (1371 visits in average). Data are presented in Fig. 1.

## Discussion

The COVID-19 pandemic is marked by a major decrease of total visits (27 PEDs in the USA from 15 March to 31 August 2020 vs a 3-year earlier comparator period with a total rate of visits down by 45.7%) [4] and an increase of the proportion of mental health-related visits (data from CDC’s National Syndromic Surveillance Program in the USA from 1 January to 7 October 2020 vs the same period in 2019) [5].

Although it is difficult to statistically compare our population with the one mentioned above, the difference observed in this large North American pediatric population seems to be consistent with our results. Indeed, the PED of the UPHN is the fourth site in France in terms of number of visits and the only one for the East PACA region which has more than one million inhabitants. The increase in mental health-related visits (44.2%) that we observed, while total visits decreased (30.0%), suggests an impact of the pandemic on children’s and adolescents’ mental health. Indeed, negative mental health effects have already been linked to home confinement among Chinese children [2] and an increased rate of suicide ideation has been observed during the pandemic among North American adolescents [6]. Recently, a stable pattern of behavioral inhibition in childhood has been shown to predict heightened anxiety in adults facing the pandemic [7].

Thus, further longitudinal studies with a developmental approach in various countries are needed to understand the underlying mechanisms of the pandemic impact on youth mental health.

## Data Availability

The data that support the findings of this study are available on request from the corresponding author.
